# Magneto-optical spectroscopy on Weyl nodes for anomalous and topological Hall effects in chiral MnGe

**DOI:** 10.1038/s41467-021-25276-1

**Published:** 2021-10-13

**Authors:** Y. Hayashi, Y. Okamura, N. Kanazawa, T. Yu, T. Koretsune, R. Arita, A. Tsukazaki, M. Ichikawa, M. Kawasaki, Y. Tokura, Y. Takahashi

**Affiliations:** 1 Department of Applied Physics and Quantum Phase Electronics Centre, University of Tokyo, Tokyo, Japan; 2 Deparment of Physics, Tohoku University, Sendai, Japan; 3 RIKEN Centre for Emergent Matter Science (CEMS), Wako, Japan; 4 Institute for Materials Research, Tohoku University, Sendai, Japan; 5 Tokyo College, University of Tokyo, Tokyo, Japan

**Keywords:** Electronic properties and materials, Topological matter

## Abstract

Physics of Weyl electrons has been attracting considerable interests and further accelerated by recent discoveries of giant anomalous Hall effect (AHE) and topological Hall effect (THE) in several magnetic systems including non-coplanar magnets with spin chirality or small-size skyrmions. These AHEs/THEs are often attributed to the intense Berry curvature generated around the Weyl nodes accompanied by band anti-crossings, yet the direct experimental evidence still remains elusive. Here, we demonstrate an essential role of the band anti-crossing for the giant AHE and THE in MnGe thin film by using the terahertz magneto-optical spectroscopy. The low-energy resonance structures around ~ 1.2 meV in the optical Hall conductivity show the enhanced AHE and THE, indicating the emergence of at least two distinct anti-crossings near the Fermi level. The theoretical analysis demonstrates that the competition of these resonances with opposite signs is a cause of the strong temperature and magnetic-field dependences of observed DC Hall conductivity. These results lead to the comprehensive understanding of the interplay among the transport phenomena, optical responses and electronic/spin structures.

## Abstract (web summary)

Previous work has proposed that the anomalous and topological Hall effects, associated with Weyl nodes, should have a signature in optical conductivity. Here, using THz optical spectroscopy, the authors assign these two effects to optical conductivity resonances, arising near band anti-crossings, in thin films of MnGe.

## Introduction

Understanding of unconventional electromagnetic phenomena stemming from the quantum topological natures is a central issue in contemporary condensed matter physics. The anomalous Hall effect (AHE) caused by spontaneous breaking of time reversal symmetry invokes a long debate regarding the microscopic origin in the past decades^1^. Several mechanisms classified into either intrinsic or extrinsic ones have been proposed so far. The intrinsic mechanism based on the band structure affected by relativistic spin-orbit coupling (SOC) was first proposed by Karplus and Luttinger, and subsequently formulated in terms of the Berry phase arising from the electronic band topology^1–5^. The enhanced Berry curvature owing to the crossing point, i.e., Weyl node^6,7^, is considered to cause the large AHE; this character is contrasted to the extrinsic origin such as skew scattering and side-jump mechanisms, which are irrelevant to the electronic band structure^8–10^. In addition to the topologically protected Weyl point, the accidental crossing points of electronic band structure also generate the Berry curvature where the SOC opens a finite gap (band anti-crossing). The subtle balance of the competition among these crossing points near the Fermi level can produce intriguing behaviors such as non-monotonic temperature dependence and giant magnitude of intrinsic AHE^11–17^. On the other hand, when the magnetic structure, to which conduction electron is coupled, is composed of noncoplanar spins with scalar spin chirality (SSC) or finite skyrmion number, the so-called topological Hall effect (THE) emerges even without SOC^18–26^. The THE is often argued in terms of emergent magnetic field in analogy to ordinary Hall effect, when the spin-chirality period is large enough compared with the lattice spacing like the conventional skyrmion-lattice phase^22^. However, the electron mean-free path exceeds the size of spin modulation period, e.g., skyrmion-lattice spacing, the THE should be considered in the momentum space in terms of Weyl electrons as in the case of AHE, rather than the real-space picture of emergent magnetic field^18,19,26^. Thus, the direct experimental evidence for the essential role of the Weyl nodes for such nontrivial behaviors of both AHE and THE remains to be experimentally elucidated.

The spectroscopic study in terms of the optical Hall conductivity will be a powerful approach to this issue. Similarly to the ordinary Hall effect arising from the cyclotron resonance, the AHE and perhaps the THE as well should exhibit the characteristic resonance structures in the optical Hall conductivity spectrum *σ*_*xy*_(*ω*)^27–32^. The interband optical transitions across each band-crossing point lead to the resonance structures in *σ*_*xy*_(*ω*), whose DC limit approaches the Hall (AH and/or TH) conductivity observed as the transport phenomena. Accordingly, the *σ*_*xy*_(*ω*) spectra quantitatively clarify the contribution from each band crossing point distinctly to AHE and THE. Since the enhanced Berry curvature of the crossing points near the Fermi level causes the large AHE and THE, the low-energy optical transition should play the essential role.

**Fig. 1:**
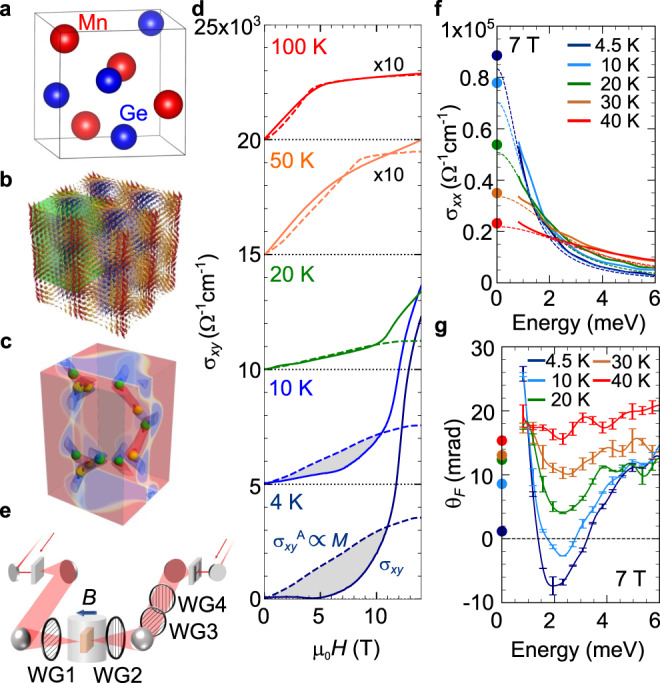
Crystal structure, magnetic structure and basic properties of MnGe thin film. **a** The crystal structure of MnGe. **b** The schematic illustration of the three-dimensional spin hedgehog lattice. **c** The positions of the emergent monopoles (green dots) and anti-monopoles (yellow dots) at zero magnetic field in the green box of panel **b**^21^. The red and blue colors represent the positive and negative emergent magnetic fields, respectively. **d** The magnetic-field (*H*) dependence of the Hall conductivity at each temperature (solid curves). The data are shifted vertically for clarity. The dashed curves (*σ*_*xy*_^A^) represent the estimated magnetic-field dependence of the anomalous Hall effect estimated from the magnetization curves (see also the discussion in Supplementary Note 1). The shaded areas, which represents the deviation from *σ*_*xy*_^A^, are ascribed mainly to topological Hall effect (THE). **e** The schematic illustration of the experimental setup. The direction of the magnetic field is parallel to the light propagation direction (Faraday configuration). WG1-4 are wire-grid polarizers. **f** The longitudinal terahertz conductivity spectra (*σ*_*xx*_(*ω*)). Filled circles at zero frequency are the DC values obtained from the transport measurement. Dashed lines are the fits with Drude formula (see the text). **g** The Faraday rotation spectra. Filled circles at zero frequency indicate the values expected from the DC transport properties (see Methods). The error bars indicate the standard deviation.

In this work, we investigate the optical transitions associated with the giant AHE and THE in MnGe thin film in terms of the terahertz optical Hall conductivity. MnGe is a member of the B20-type alloys with the chiral crystal structure (Fig. 1a) and below 170 K exhibits the helimagnetically ordered state, which is viewed as composed of the hybridized three orthogonal spin helices with helical pitch of 3 nm to form spin hedgehog-antihedgehog lattice^33–36^, as shown in Fig. 1b. Each hedgehog and antihedgehog behave as emergent magnetic monopole and anti-monopole acting on the conduction electron, which are bridged over by skyrmion strings, as shown in Fig. 1c. When external magnetic field is applied on this hedgehog lattice, the bridging skyrmion-strings are elongated or shrunk, thereby producing emergent magnetic field or non-zero skyrmion number density in total and leading to the large THE in the magnetic-field range in between the zero-field state and the field-aligned ferromagnetic state^35^. On the other hand, the inherently large SOC in this compound also produces the conventional AHE approximately in proportion to the field-induced magnetization. Therefore, MnGe provides a good arena to spectroscopically identify and distinguish the Berry curvatures generated around the Weyl nodes by the SOC as well as by the SSC (skyrmion number), which respectively show up as AHE and THE. We demonstrate that the terahertz resonances of the band anti-crossing points (Weyl nodes) close to the Fermi level are assigned distinctly to the origins of AHE and THE and that the competition of these resonances causes the strong temperature and magnetic-field dependence of DC Hall conductivity as observed.

## Results

### Magento-transport properties

Figure 1d summarizes the Hall conductivity *σ*_*xy*_ of the MnGe thin film used in this study when magnetic field *H* is applied normal to the film plane, i.e., *H||*[111]. At relatively high temperatures, e.g., above 30 K, *σ*_*xy*_ increases with *H*, followed by the saturation, which well corresponds to the magnetization curve as a hallmark of AHE (dashed lines in Fig. 1d; see also the discussion in Supplementary Note 1). With decreasing temperature below 30 K, however, the magnetic-field dependence of *σ*_*xy*_ deviates from that of the conventional AHE being proportional to the magnetization; this is due to the increased negative component of *σ*_*xy*_, as indicated by shaded regions in Fig. 1d. This dip-like structure has been ascribed to the THE^21,33,35,36^, which is expected to appear in the presence of the SSC of the noncoplanar spin texture. More specifically, the THE in MnGe originates from the three-dimensional topological spin crystal consisting of the hedgehog and antihedgehog spin textures (Fig. 1b)^33–36^. These spin textures act as monopole and anti-monopole of the emergent magnetic field which is canceled out in total at zero external magnetic field. Upon the application of external magnetic field, however, the emergent monopole and anti-monopole are forced to move in opposite directions; these movements of emergent magnetic monopoles/anti-monopoles are accompanied by deformation of the bridging skyrmion-strings (Fig. 1c), giving rise to the uncancelled emergent magnetic field or the relevant Berry-curvature generation in the momentum space. We ascribe these topological magnetic orders to the origin of THE with negative *σ*_*xy*_ values, which show a negative peak around a middle field between the zero-field state and the forced ferromagnetic transition^21,33,35,36^.

### Terahertz polarimetry

We performed the phase sensitive time-domain terahertz polarimetry in the normal incidence for the MnGe(111) thin film with the thickness of 85 nm fabricated on the insulating Si(111) substrate (see also Methods). We obtained the complex spectra of the magneto-optical Faraday rotation in the magnetic field applied along the MnGe [111] axis, i.e., normal to the film plane (Fig. 1e). We note that the hedgehog lattice is sensitive to the magnetic anisotropy depending on the film thickness and deformed in the thin film form^36^. The three *q* vectors i.e., the magnetic basis vectors of hedgehog lattice state, tilt toward the normal direction in the thin film; accordingly, the hedgehog and antihedgehog are sparsely distributed as compared with the bulk system. The dataset obtained by this technique directly provides the optical Hall conductivity *σ*_*xy*_(*ω*) in addition to the longitudinal optical conductivity *σ*_*xx*_(*ω*) (see Methods). The real part of *σ*_*xx*_(*ω*) shows a steep increase towards zero frequency at low temperatures (Fig. 1f). In the terahertz region, the *σ*_*xx*_(*ω*) spectra represent the conduction electron dynamics as expressed by the Drude model ${\sigma }_{{xx}}\left(\omega \right)=\frac{{\varepsilon }_{0}{\omega }_{p}^{2}\tau }{1-i\omega \tau }$; *ε*_0_, 1/*τ* and *ω*_p_ are the dielectric constant in vacuum, scattering rate and plasma frequency, respectively. The scattering rate 1/*τ*, i.e., the width of the zero-frequency peak in Re *σ*_*xx*_(*ω*), decreases monotonically with decreasing temperature and reaches ~ 1 meV at 4.5 K, indicating good metallic conduction and high quality of the sample (Supplementary Fig. 4).

**Fig. 2:**
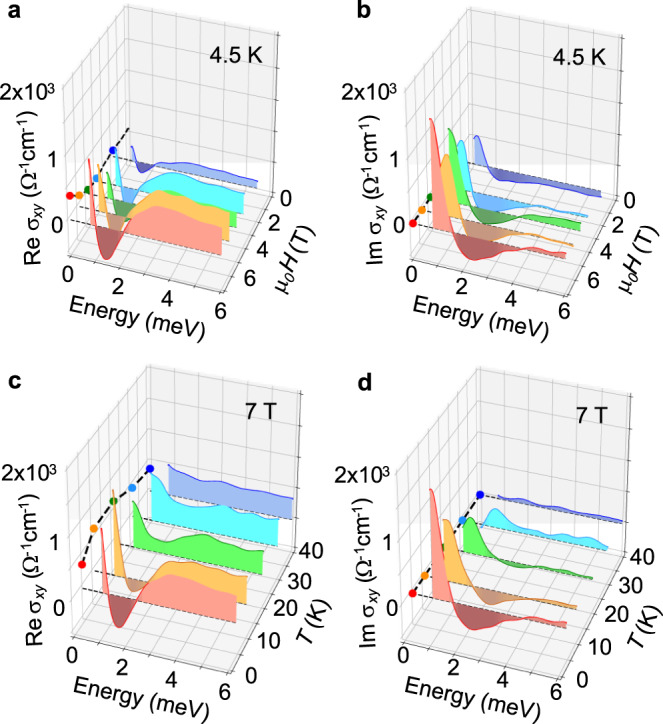
Terahertz Hall conductivity spectra of MnGe thin film. **a**, **b** The magnetic-field dependence of the real (**a**) and imaginary (**b**) parts of the Hall conductivity spectra (*σ*_*xy*_(*ω*)) at 4.5 K. The spectra are measured at 2, 4, 5, 6, and 7 T. **c**, **d** The temperature dependence of the real (**c**) and imaginary (**d**) parts of the Hall conductivity spectra at 7 T. The spectra are measured at 4.5, 10, 20, 30, and 40 K. Filled circles at zero frequency are the DC values acquired from the transport measurement.

In contrast to the conventional metallic behavior observed in the *σ*_*xx*_(*ω*) spectra, the Faraday rotation and Hall conductivity spectra show prominent features at low temperatures and at moderate magnetic fields. At 40 K and 7 T, the Faraday rotation spectrum *θ*_F_(*ω*) is almost constant, and the magnitude is consistent with the DC Hall angle (a red curve in Fig. 1g). With decreasing temperature, the dip-like resonance structure develops at ~ 2.0 meV, and shows a sign reversal at 4.5 and 10 K. To examine the correlation with the AHE and THE quantitatively, the complex optical Hall conductivity spectra, Re *σ*_*xy*_(*ω*) and Im *σ*_*xy*_(*ω*), are deduced from *σ*_*xx*_(*ω*), *θ*_F_(*ω*) and ellipticity *η*_F_(*ω*) (see Methods). The dramatic spectral changes in the Hall conductivity spectra *σ*_*xy*_(*ω*) are observed with the changes of magnetic field (Fig. 2a, b) and temperature (Fig. 2c, d). In the course of increasing the magnetic field at 4.5 K, the sharp dip structure at ~1.5 meV develops in addition to the increase of the flat background above 3 meV in Re *σ*_*xy*_(*ω*) (Fig. 2a). On the other hand, the Im *σ*_*xy*_(*ω*) shows a shallow dip at ~2.3 meV and the steep rise towards lower frequency, which are enhanced at higher magnetic fields (Fig. 2b). Since the Im *σ*_*xy*_(*ω*) should converge to zero at *ω* = 0 due to the causality constraint (see *ω* = 0 plane in Fig. 2b), the emergence of the peak structure below 1 meV is suggested in high magnetic fields.

The DC Hall conductivity at 7 T shows the anomaly at low temperatures (the filled circles at the *ω* = 0 plane in Fig. 2c); it increases up to 680 Ω^−1^cm^−1^ at 10 K from 380 Ω^−1^cm^−1^ at 40 K with decreasing temperature, which is followed by the decrease towards the lowest temperature (4.5 K). This low-temperature anomaly in DC Hall conductivity coincides with the appearance of the resonance structure in *σ*_*xy*_(*ω*) (Fig. 2c, d). At 40 K, both Re *σ*_*xy*_(*ω*) and Im *σ*_*xy*_(*ω*) are flat and consistent with the DC Hall conductivity (7 T; blue curves). With decreasing temperature, the resonance structures rapidly develop while keeping *σ*_*xy*_(*ω*) above 4 meV almost unchanged. It is thus concluded that the dramatic change of transport Hall conductivity at low temperatures and moderate magnetic fields (at least up to 7 T) stems from the resonance structures in terahertz region (< 4 meV). These low-energy resonances in *σ*_*xy*_(*ω*) indicate the interband optical transitions across the band (anti-)crossing points or Weyl nodes, which suggests the dominant role of the Berry-curvature mechanism for the both AHE and THE in MnGe. We note that the ordinary Hall effect hardly contributes to the *σ*_*xy*_(*ω*) in this energy region (see the discussion in Supplementary Note 2).

### Analysis model for Hall conductivity spectrum

**Fig. 3:**
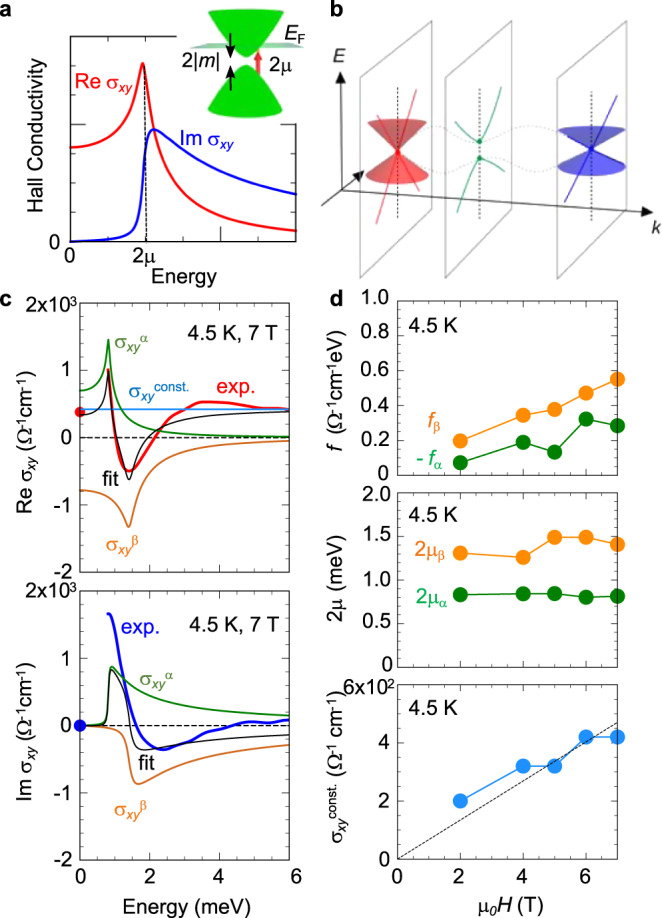
Two-band model analysis for terahertz resonances. **a** The AH conductivity spectra calculated from the two-band model. (inset) The schematic illustration of the single anti-crossing point. **b** Schematic illustration of the electronic structure considered in the present analysis. **c** The red (Re *σ*_*xy*_) and blue (Im *σ*_*xy*_) curves show experimental results and the black curves represent the fitting curves at 4.5 K and 7 T. The *σ*_*xy*_^α^ (green) and *σ*_*xy*_^β^ (orange) are optical Hall conductivity spectra arising from *α*- and *β*-bands, respectively. The constant term *σ*_*xy*_^const.^ (light blue line) represents the lower-lying tail of the higher-energy resonances. The red and blue circles at zero frequency are the Hall conductivity acquired from transport measurement. **d** The magnetic-field dependence of the fitting parameters. *f*_*α,β*_, 2*μ*_*α,β*_ and *σ*_*xy*_^const.^ are the spectral weights related to the anti-crossing gap *m*_α,β_, resonance energies and constant term, respectively. After we determine *δ*_*α*_ = 0.06 meV and *δ*_*β*_ = 0.53 meV using the data at 7 T, we use the same *δ*_*α*_ and *δ*_*β*_ for other magnetic fields. The dashed line in the lower panel represents the guide to the eyes.

To understand the spectral feature of the terahertz resonances, we introduce the two-band model describing the intrinsic AHE and THE with minimal assumptions (Fig. 3a): The model Hamiltonian *H*(*k*) is expressed as follows^27,28,30^;

1 $$\begin{array}{c}H\left(k\right)=-\mu {\sigma }_{0}+\mathop{\sum} _{i=x,y,z}{h}_{i}\left(k\right){\sigma }_{i},\end{array}$$

where *σ*_0_ and *σ*_*i*_ (*i* = *x*, *y*, *z*) are the identity and Pauli matrices, respectively, and *μ* is the chemical potential measured from the band crossing point. Accordingly, we assume that the band dispersion is quasi two-dimensional and (*h*_*x*_*, h*_*y*_*, h*_*z*_) = (*k*_*x*_*, k*_*y*_*, m*), so that it has the level splitting of 2|*m*| at the crossing point (Fig. 3a, inset). We calculate the optical Hall conductivity *σ*_*xy*_(*ω*) with use of the general expression given by the Kubo formula;

2 $$\begin{array}{c}{\sigma }_{{xy}}\left(\omega \right)=i\mathop{\sum} _{n,m}\frac{f\left({\varepsilon }_{m}\right)-f\left({\varepsilon }_{n}\right)}{{\varepsilon }_{m}-{\varepsilon }_{n}}\frac{\langle m|{J}_{y}|n\rangle \langle n|{J}_{x}|m\rangle }{\omega +i\delta +{\varepsilon }_{m}-{\varepsilon }_{n}},\end{array}$$

where the *J*_*x*(*y*)_ is the current operator given by $\frac{\hslash }{e}\mathop{\sum}\limits_{k}{c}^{{{\dagger}} }(k)\frac{\partial H\left(k\right)}{\partial {k}_{x\left(y\right)}}c(k)$. *f*(*ε*_n_) is the Fermi distribution function, ${\varepsilon }_{n}$ and |*n*〉 are the energy and the Bloch wave function of the *n*th band, respectively, and *δ* is the phenomenological damping constant. *c*(*k*) (${c}^{{{\dagger}} }$(*k*)) is the annihilation (creation) operator. The energy-dependent Hall conductivity *σ*_*xy*_(*ω*) is thus given by^27,28^;

3 $$\begin{array}{c}{\sigma }_{{xy}}\left(\omega \right)=\frac{{e}^{2}}{2{ha}}\frac{m}{{{\hslash }}\omega +i\delta }{\ln}\left|\frac{-{{\hslash }}{{\omega }}-i{{\delta }}+2{{\mu }}}{{{\hslash }}{{\omega }}+i{{\delta }}+2{{{\mu }}}}\right|,\end{array}$$

where *e*, *h* and *a* are the elementary electric charge, Planck constant and lattice constant, respectively. Re *σ*_*xy*_(*ω*) and Im *σ*_*xy*_(*ω*) in Eq. (3) have sharp resonance peaks at the energy of 2*μ* without the sign change as shown in Fig. 3a. Since the observed spectra show the sign change for both of Re *σ*_*xy*_(*ω*) and Im *σ*_*xy*_(*ω*) (Fig. 2), it is reasonable to assume two resonances with opposite signs of *m*. This assumption is consistent with the coexistence of AHE and THE with opposite signs in the DC Hall conductivity (Fig. 1d). Accordingly, the total Hall conductivity spectrum consisting of the vertical transitions on two anti-crossing points near the Fermi level is expressed as,

4 $${\sigma }_{{xy}}\left(\omega \right)	= \;{{\sigma }_{{xy}}}^{\alpha }\left(\omega \right)+{{\sigma }_{{xy}}}^{\beta }\left(\omega \right)+{{\sigma }_{{xy}}}^{{const}.}\\ 	 = \;\mathop{\sum} _{i=\alpha ,\beta }\frac{{e}^{2}}{2{ha}}\frac{{m}_{i}}{{{\hslash }}\omega +i\delta }{{{{{\rm{ln}}}}}}\left|\frac{-{{\hslash }}\omega -i{\delta }_{i}+2{\mu }_{i}}{{{\hslash }}\omega +i{\delta }_{i}+2{\mu }_{i}}\right|+{{\sigma }_{{xy}}}^{{const}.}\\ 	 \equiv \; \mathop{\sum} _{i=\alpha ,\beta }\frac{{f}_{i}}{{{\hslash }}\omega +i{\delta }_{i}}{{{{{\rm{ln}}}}}}\left|\frac{-{{\hslash }}\omega -i{\delta }_{i}+2{\mu }_{i}}{{{\hslash }}\omega +i{\delta }_{i}+2{\mu }_{i}}\right|+{{\sigma }_{{xy}}}^{{const}.},$$

where *α* and *β* are band indices. Two anti-crossing points of *α-* and *β*-bands give rise to *σ*_*xy*_^α^(*ω*) and *σ*_*xy*_^β^(*ω*), respectively. The constant term *σ*_*xy*_^const.^ represents the low-energy tail of higher-energy resonances above the present energy window. We note that in the chiral metal MnGe the band crossing with intense Berry curvature is the (three-dimensional) Weyl point, which is robust against some perturbation^6,7^; the minimal Hamiltonian is given by, $H=\mathop{\sum}\limits_{i=x,y,z}{v}_{i}{k}_{i}{\sigma }_{i}$. In the present model analysis, we consider the anti-crossing points present along the certain *k* vector interconnecting two Weyl points (Fig. 3b). Here, we introduced *f*_*i*_ as a free parameter representing the spectral weight in Eq. (4), which effectively describes the averaged gap magnitude; the *δ* represents the spectral width of the resonance, which can also reflect the three-dimensional band dispersion (Supplementary Note 3). In the results shown below, we adopted the fitting parameters producing the DC limit value less than the typical upper limit of the intrinsic AH conductivity^37^, $\frac{{e}^{2}}{{ha}}$ ~1000 Ω^−1^cm^−1^. As shown in Fig. 3c, the calculated spectra (black), which are composed of *σ*_*xy*_^α^(*ω*) (green), *σ*_*xy*_^β^(*ω*) (orange) and *σ*_*xy*_^const.^ (light blue), quantitatively reproduces the experimental spectral features; the dispersive shape with the sign change in Re *σ*_*xy*_(*ω*) (red) and the large positive peak (~1 meV) in Im *σ*_*xy*_(*ω*) (blue). The similar results were obtained for the spectra at other magnetic fields and temperatures. The fitting parameters at 4.5 K are summarized in Fig. 3d. The spectral weight *f*_*α,β*_ and *σ*_*xy*_^const.^ monotonically increase by applying the magnetic field, while the resonance energies of *α*-band (2*μ*_α_ ~ 0.8 meV) and *β*-band (2*μ*_β_ ~ 1.4 meV) show little magnetic-field dependence. These small energy scales of resonance energies (1 meV ~ 11.6 K) are consistent with the strong temperature dependence of the Hall responses below 30 K, because the low-energy structures should be smeared out by thermal agitations at high temperatures.

### Comparison with transport results

**Fig. 4:**
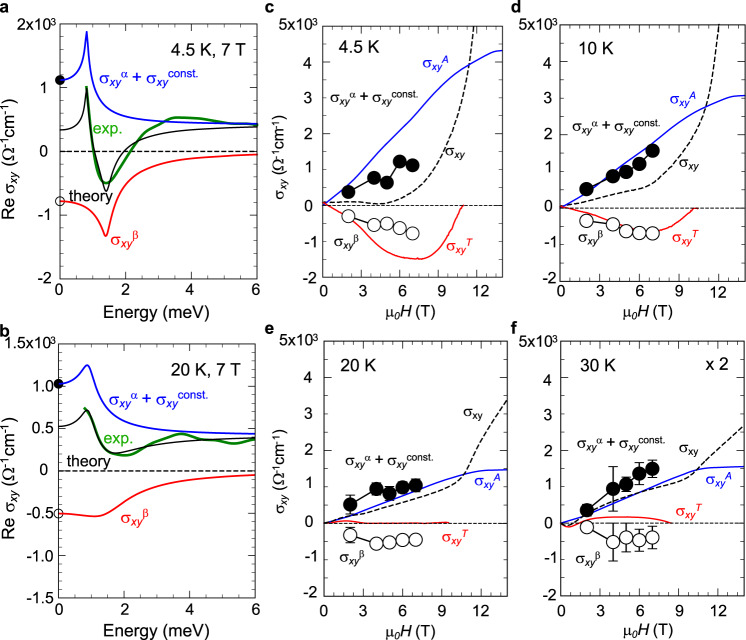
Anomalous Hall effect (AHE) and topological Hall effect (THE) obtained from terahertz and transport measurements. **a**, **b** The decomposition of the experimental spectra into two resonances corresponding to the AHE (blue) and THE (red) at (**a**) 4.5 K and (**b**) 20 K. The experimental and theoretical spectra are shown by green and black curves, respectively. These spectra for 4.5 K are multiplied by a factor of two. **c**–**f** The magnetic-field dependence of the AHE (Re{*σ*_*xy*_^α^ + *σ*_*xy*_^const.^}, filled circle) and THE (Re{*σ*_*xy*_^β^}, open circle) at *ω* = 0 obtained from the analysis for terahertz spectra; (**c)** 4.5 K, (**d)** 10 K, (**e)** 20 K, and (**f)** 30 K. The zero-frequency limit (*ω* = 0) of the AHE (*σ*_*xy*_^α^ + *σ*_*xy*_^const.^) and THE (*σ*_*xy*_^β^) are displayed by filled and open circles also in **a**, respectively. The red and blue curves in **c**–**f** show the AH conductivity *σ*_*xy*_^A^ and TH conductivity *σ*_*xy*_^T^ deduced from the transport measurement, respectively. The total Hall conductivity is also shown by dotted curves. The decomposition of AHE and THE is possible in a relatively low field region (≤ 7 T) as exemplified in early works^21,33,36^, whereas the steep increase of *σ*_*xy*_ at low temperatures and above 12 T in a field-aligned ferromagnetic region is supposed to stem from the spin-chirality skew scattering^38–40^ (see also Supplementary Note 1).

The signs of the DC AH and TH conductivities are observed to be positive and negative, respectively (Fig. 1d). The terahertz Hall conductivity spectra reveal the presence of two positive components, *σ*_*xy*_^α^(*ω*) and *σ*_*xy*_^const.^, and negative one, *σ*_*xy*_^β^(*ω*). Therefore, the negative resonance, *σ*_*xy*_^β^(*ω*), can be ascribed to the THE, while the positive resonance *σ*_*xy*_^α^(*ω*) to the positive AHE. The positive *σ*_*xy*_^const.^ may possibly have both contributions from the AHE and THE whose resonances locate at higher energies, but appears to be mostly dominated by the AHE process in the present case because of its nearly *M*(*H*)-linear behavior (lower panel of Fig. 3d). These assignments are further verified by the quantitative comparison with the transport measurement. We calculated the DC-limit values of these resonances, i.e., Re{*σ*_*xy*_^α^(*ω* *=* 0)+*σ*_*xy*_^const.^} and Re{*σ*_*xy*_^β^(*ω* = 0)} as indicated by filled and open circles at 4.5 K (Fig. 4a) and 20 K (Fig. 4b), and plotted as functions of magnetic field for each temperature in Fig. 4c–f. The *σ*_*xy*_^α^(*ω* = 0) + *σ*_*xy*_^const.^ (filled circles) quantitatively reproduces the monotonic increase of the AHE estimated from the transport data (a blue line; see also Supplementary Note 1) with increasing the magnetic field at each temperature. The enhancement of the magnitude of the estimated THE (red line) as a function of magnetic field is also described by the *σ*_*xy*_^β^(*ω* = 0) (open circles). In addition, the rapid diminishment of the THE at higher temperatures (*T* = 20, 30 K) is consistent with the *σ*_*xy*_^β^(*ω* = 0) obtained from the terahertz measurement. These observations confirm the whole consistency of our theoretical analysis and assignment, thus demonstrating that the emergence and competition of these two anti-crossing points are a major origin of the strong magnetic-field dependence and temperature dependence of DC Hall conductivity.

## Discussion

It should be emphasized that the observation of the resonance of the THE indicates that the formation of the topological spin texture modifies the electronic band structure. For MnGe thin film, the magnetic period (~3 nm) is shorter than the mean-free path (~3.6 nm at 2 K) estimated from the simple free-electron model (Supplementary Note 4). In this regime, the noncoplanar spin texture with SSC, as exemplified by the ordered skyrmion strings bridging the emergent magnetic monopoles and anti-monopoles (Fig. 1c), can induce the anti-crossing points in the band dispersions, which gives rise to the THE as reported for pyrochlore Mo-oxides^18,19,26^. In such the short-period spin order with SSC, the THE is ascribed to the Berry curvature arising from the band crossings similarly to AHE, while the AHE and THE can be associated with different band crossings as suggested by the recent study^26^; the Berry curvature for the AHE arises from the band crossings composed of the equal- and opposite-spin band pairs impartially while that for the THE only of the opposite-spin band pairs. This momentum-space picture of the THE is not applicable to the archetypal skyrmion-hosting material MnSi with much longer magnetic period (~18 nm). In MnSi, the magnitude of the THE, that is much smaller than the present case, is well explained in terms of the real-space picture that the conduction electrons are deflected by the emergent magnetic field carried by skyrmions^20,22,23^; namely, there is no necessity to invoke the momentum-space Weyl nodes.

We note that the presence of lots of Weyl nodes can be confirmed by the density functional theory calculation for the forced ferromagnetic state (Supplementary Fig. 6). We also calculate the optical Hall conductivity spectra. The sharp resonance structure with positive sign can show up at certain Fermi level (Supplementary Fig. 7), which well captures the spectral characteristics of the terahertz resonance producing the AHE. Nevertheless, it is difficult to directly compare these calculated spectra for the ferromagnetic state with the experimental one for the hedgehog lattice because the band structure may be significantly modified by the band folding due to the formation of the short-period topological spin texture. Such a reconstructed band structure probably changes the AHE features of Berry curvature origin. The clear elucidation of the modified band structure by the formation of small-size skyrmions will be the important future task.

In conclusion, we have investigated the low-energy electron dynamics associated with the AHE and THE in MnGe thin film. We have found that the prominent low-energy resonance structures unveiled by the terahertz Hall conductivity spectra dominantly contribute to the DC Hall conductivity. This observation experimentally demonstrates the band anti-crossing points or Weyl nodes just above or beneath the Fermi level indeed realize the large AHE and THE as anticipated. Moreover, spectral characteristics of optical Hall conductivity clearly point out that the competition of low-energy anti-crossing points causes the strong temperature and magnetic-field dependence of the DC Hall conductivity. The present terahertz magneto-optical spectroscopy establishes the Berry-curvature generation in the electronic bands, relevant to AHE as well as THE with short-period SSC, which will further stimulate exploration of giant or even quantized AHE/THE^41,42^ and promote understanding of topological physics and promising functions.

## Methods

### Thin film growth

An 85-nm-thick MnGe thin film was fabricated on Si(111) substrates with use of the molecular beam epitaxy technique. The details of the growth procedure and characterization are described in Supplementary Note 6.

### Transport measurements

The magneto-resistivity and Hall resistivity were measured by using Physical Property Measurement System (Quantum Design). Concerning the analysis for the decomposition of the AHE and THE, see Supplementary Note 1.

### Terahertz time domain spectroscopy (THz-TDS)

In the THz-TDS, laser pulses with the duration of 100 fs from a mode-locked Ti: sapphire laser were split into two paths to generate and detect THz pulses with using the photoconductive antenna. Transmittance spectra of the thin film were obtained by measuring the transmission of both the sample and bare substrate. We used the following standard formula to obtain the complex conductivity *σ*_*xx*_(*ω*) = Re *σ*_*xx*_(*ω*) + *i* Im *σ*_*xx*_(*ω*) of the thin film;

5 $$\begin{array}{c}t\left({{{{{\rm{\omega }}}}}}\right)=\frac{1+{n}_{s}}{1+{n}_{s}+{Z}_{0}d{{{{{{\rm{\sigma }}}}}}}_{{xx}}\left({{{{{\rm{\omega }}}}}}\right)},\end{array}$$

where *t* (*ω*), *d*, *Z*_0_ and *n*_s_ are the complex transmittance, the thickness of the film, the impedance of free space (377 Ω), and the refractive index of the Si substrate, respectively.

### Faraday rotation measurements

The rotatory component of the transmitted THz pulses *E*_*y*_(*t*), which is polarized perpendicular to the incident light *E*_*x*_(*t*), was measured in the crossed-Nicols configuration by using wire-grid polarizers. To eliminate the background signal, we anti-symmetrized *E*_*y*_(*t*) with respect to positive and negative magnetic fields. The Fourier transformation of the THz pulses *E*_*x*_(*t*) and *E*_*y*_(*t*) gives the Faraday rotation *θ*_F_(*ω*) and ellipticity *η*_F_(*ω*); *E*_*y*_(*ω*)/*E*_*x*_(*ω*) ≃ *θ*_F_(*ω*) + *iη*_F_(*ω*) for the small rotation angles.

### Terahertz Hall conductivity

By using the longitudinal conductivity spectra *σ*_*xx*_(*ω*) and Faraday rotation and ellipticity spectra *θ*_F_(*ω*) + *iη*_F_(*ω*), the Hall conductivity spectra were calculated from the following formula; ${\sigma }_{xy}(\omega)=(\theta_{{{{{\rm{F}}}}}}+i\eta _{{{{{\rm{F}}}}}})\frac{1+{n}_{s}+{Z}_{0}d{\sigma }_{xx}(\omega)}{{Z}_{0}d}$ ^28^. To estimate the DC-limit rotation angle *θ*_F_(*ω* = 0) in Fig. 1g, we used this formula by substituting *σ*_*xx*_(0) and *σ*_*xy*_(0) obtained from the transport measurements.

## Supplementary information

Supplementary Information

## Data Availability

The data that support the plots of this study are available from the corresponding author upon reasonable request.

## References

[1] Nagaosa N, Sinova J, Onoda S, MacDonald AH, Ong NP (2010). Anomalous Hall effect. Rev. Mod. Phys..

[2] Karplus R, Luttinger JM (1954). Hall effect in ferromagnetics. Phys. Rev..

[3] Onoda M, Nagaosa N (2002). Topological nature of anomalous Hall effect in ferromagnets. J. Phys. Soc. Jpn..

[4] Onoda S, Sugimoto N, Nagaosa N (2008). Quantum transport theory of anomalous electric, thermoelectric, and thermal Hall effects in ferromagnets. Phys. Rev. B.

[5] Xiao D, Chang M-C, Niu Q (2010). Berry phase effects on electric properties. Rev. Mod. Phys..

[6] Yan B, Felser C (2017). Topological materials: Weyl semimetals. Annu. Rev. Condens. Matter Phys..

[7] Armitage NP (2018). Weyl and Dirac semimetals in three-dimensional solids. Rev. Mod. Phys..

[8] Smit J (1955). The spontaneous Hall effect in ferromagnetics I. Physica.

[9] Smit J (1958). The spontaneous Hall effect in ferromagnetics II. Physica.

[10] Berger L (1970). Side-jump mechanism for the Hall effect of ferromagnets. Phys. Rev. B.

[11] Fang Z (2003). The anomalous Hall effect and magnetic monopoles in momentum space. Science.

[12] Lee W-L, Watauchi S, Mirror VL, Cava RJ, Ong NP (2004). Dissipationless anomalous Hall current in the ferromagnetic spinel CuCr2Se4-xBrx. Science.

[13] Nakatsuji S, Kiyohara N, Higo T (2015). Large anomalous Hall effect in a non-collinear antiferromagnet at room temperature. Nature.

[14] Suzuki T (2016). Large anomalous Hall effect in a half-Heusler antiferromagnet. Nat. Phys..

[15] Ye L (2018). Massive Dirac fermions in a ferromagnetic kagome metal. Nature.

[16] Ghimire NJ (2018). Large anomalous Hall effect in the chiral-lattice antiferromagnet CoNb3S6. Nat. Commun..

[17] Liu E (2018). Giant anomalous Hall effect in a ferromagnetic kagome-lattice semimetal. Nat. Phys..

[18] Ohgushi K, Murakami S, Nagaosa N (2000). Spin anisotropy and quantum Hall effect in the kagome lattice: chiral spin state based on a ferromagnet. Phys. Rev. B.

[19] Taguchi Y, Oohara Y, Yoshizawa H, Nagaosa N, Tokura Y (2001). Spin chirality, Berry phase, and anomalous Hall effect in a frustrated ferromagnet. Science.

[20] Neubauer A (2009). Topological Hall effect in the A-phase of MnSi. Phys. Rev. Lett..

[21] Kanazawa N (2011). Large topological Hall effect in a short-period helimagnet MnGe. Phys. Rev. Lett..

[22] Nagaosa N, Tokura Y (2013). Topological properties and dynamics of magnetic skyrmions. Nat. Nanotechnol..

[23] Franz C (2014). Real-space and reciprocal-space Berry phases in the Hall effect of Mn1-xFexSi. Phys. Rev. Lett..

[24] Kurumaji T (2019). Skyrmion lattice with a giant topological Hall effect in a frustrated triangular-lattice magnet. Science.

[25] Hirschberger M (2020). Topological Nernst effect of the two-dimensional skyrmion lattice. Phys. Rev. Lett..

[26] Hirschberger M (2021). Geometrical Hall effect and momentum-space Berry curvature from spin-reversed band pairs. Phys. Rev. B.

[27] Iguchi S (2009). Optical probe for anomalous Hall resonance in ferromagnets with spin chirality. Phys. Rev. Lett..

[28] Shimano R (2011). Terahertz Faraday rotation induced by an anomalous Hall effect in the itinerant ferromagnet SrRuO3. Europhys. Lett..

[29] Kim M-H (2013). Infrared anomalous Hall effect in CaxSr1-xRuO3 films. Phys. Rev. B.

[30] Tse W-K, MacDonald AH (2010). Giant magneto-optical Kerr effect and universal Faraday effect in thin-film topological insulators. Phys. Rev. Lett..

[31] Tse W-K, MacDonald AH (2010). Magneto-optical and magnetoelectric effects of topological insulators in quantizing magnetic fields. Phys. Rev. B.

[32] Aguilar RV (2012). Terahertz response and colossal Kerr rotation from the surface states of the topological insulator Bi2Se3. Phys. Rev. Lett..

[33] Kanazawa N (2012). Possible skyrmion-lattice ground state in the B20 chiral-lattice magnet as seen via small-angle neutron scattering. Phys. Rev. B.

[34] Tanigaki T (2015). Real-space observation of short-period cubic lattice of skyrmions in MnGe. Nano Lett..

[35] Kanazawa N (2016). Critical phenomena of emergent magnetic monopoles in a chiral magnet. Nat. Commun..

[36] Kanazawa N (2017). Topological spin-hedgehog crystals of a chiral magnet as engineered with magnetic anisotropy. Phys. Rev. B.

[37] Burkov AA (2014). Anomalous Hall effect in Weyl metals. Phys. Rev. Lett..

[38] Ishizuka H, Nagaosa N (2018). Spin chirality induced skew scattering and anomalous Hall effect in chiral magnets. Sci. Adv..

[39] Kanazawa N (2020). Direct observation of the statics and dynamics of emergent magnetic monopoles in a chiral magnet. Phys. Rev. Lett..

[40] Fujishiro Y (2021). Giant anomalous Hall effect from spin-chirality scattering in a chiral magnet. Nat. Commun..

[41] Chang CZ (2013). Experimental observation of the quantum anomalous Hall effect in a magnetic topological insulator. Science.

[42] Hamamoto K, Ezawa M, Nagaosa N (2015). Quantized topological Hall effect in skyrmion crystal. Phys. Rev. B.

