# Supplementary material for: Magneto-optical spectroscopy on Weyl nodes for anomalous and topological Hall effects in chiral MnGe
Source: Nat Commun. 2021 Oct 13;12:5974. doi: 10.1038/s41467-021-25276-1 (PMC8514541; doi:10.1038/s41467-021-25276-1)
Supplement: Supplementary file 1 — Supplementary Information [file 41467_2021_25276_MOESM1_ESM.pdf]

Supplementary Information for
Magneto-optical spectroscopy on Weyl nodes for anomalous and topological Hall effects in chiral MnGe

Y. Hayashi[1*, †], Y. Okamura[1*, †], N. Kanazawa[1*], T. Yu[1], T. Koretsune[2], R. Arita[1,3], A. Tsukazaki[4], M. Ichikawa[1], M. Kawasaki[1,3], Y. Tokura[1,3,5] and Y. Takahashi[1,3†]

[1]*Department of Applied Physics and Quantum Phase Electronics Centre, University of Tokyo, Tokyo 113-8656, Japan*

[2]*Deparment of Physics, Tohoku University, Sendai 980-8578, Japan*

[3]*RIKEN Centre for Emergent Matter Science (CEMS), Wako 351-0198, Japan*

[4]*Institute for Materials Research, Tohoku University, Sendai 980-8577, Japan*

[5]*Tokyo College, University of Tokyo, Tokyo 113-8656, Japan*

[*] These authors equally contributed to this work.

[†] To whom correspondence should be addressed (hayashi@ce.t.u-tokyo.ac.jp, okamura@ap.t.u-tokyo.ac.jp, youtarou-takahashi@ap.t.u-tokyo.ac.jp)

Supplementary Note 1: Analysis of the anomalous and topological Hall effects.

At high temperatures (above 30 K), the magnetic-field dependence of the Hall conductivity can be well described by combination of the ordinary and anomalous Hall effects. To evaluate each contribution, we used the following formula,

$$\sigma_{xy} = R_0\sigma_{xx}^2 B + S_\mathrm{A} M. \qquad (1)$$

The first and second terms of the right-hand side represent the ordinary and anomalous Hall conductivities, respectively. R_0 and S_A are the ordinary and anomalous Hall coefficients, respectively[S1]. With using the *M-H* curves (Supplementary Fig. 1), the experimental data can be well fitted solely and we obtained R_0 = -3.52 nΩ cm/T at 100 K. S_A is also deduced in Supplementary Fig. 2, which steeply increases towards low temperature regions as discussed in the main text. Here, because of the difficulty in measuring the film magnetization up to 14 T, as for the data below 100 K, we used the *M-H* curve of the bulk poly crystal calibrated by the demagnetization field[S3] which shows the quantitatively similar behavior at least up to 7 T (Supplementary Fig. 1).

With lowering the temperatures, the Hall conductivity deviates from the conventional AHE described above due to the emergence of the THE. Furthermore, it increases sharply even above 12 T in a field-aligned ferromagnetic region. The observed value far exceeds the possible upper limit (~ 10^3 S/cm) of the intrinsic AH conductivity based on the Berry curvature from Weyl nodes; this large value has recently been assigned to the extrinsic skew scattering by the spin-chirality cluster state, i.e., spin-chirality skew scattering in the ferromagnetic background[S4-S6], but this is not relevant to the Weyl physics discussed here.

Below we discuss the decomposition of the Hall conductivity into those four contributions, OHE, AHE, THE and spin-chirality skew scattering at low temperatures.

Since the contribution from the spin-chirality skew scattering is much larger than that from the typical intrinsic AHE near the H_C in the thin film[S4], it is important to carefully treat the spin-chirality skew scattering in estimating the anomalous Hall coefficient S_A. To this end, we calculate the S_A at the magnetic field H_C^* where the steep increase of σ_{xy} arising from the spin-chirality skew scattering just starts. We assume that the Hall conductivity is almost dominated by intrinsic AHE at H_C^*, which is defined as follows. After subtracting the ordinary Hall contribution, we find the clear peak anomaly in $d\sigma_{xy}/dH$ corresponding to the steep increase of σ_{xy} due to the spin-chirality skew scattering. The peak anomaly can be quantified by the Gaussian fitting (dotted curve in Supplementary Fig. 3a), whose integral gives the rough approximation of the spin-chirality skew scattering (green curve in Supplementary Fig. 3b). $d\sigma_{xy}/dH$ deviates from the Gaussian fitting at the certain magnetic field of 11.0 T, which can be considered as the departure of the steep increase, i.e., H_C^*. Eventually, by subtracting the contribution from the spin-chirality skew scattering, we determine the anomalous Hall coefficient S_A so as to satisfy $\sigma_{xy}(H_C^*) - R_0(100K)\sigma_{xx}^2 H = S_A M(H_C^*)$ and calculate the AHE (red curve in Supplementary Fig. 3b; see also Supplementary Fig. 2). The remaining component is assigned to the THE (blue curve in Supplementary Fig. 3b). The fluctuation-driven spin-chirality skew scattering should be enhanced near the H_C in reality, however, the AH and TH conductivities can be estimated appropriately as far as the moderate-field regime ($\leq$ 7 T) is concerned.

Supplementary Note 2: Ordinary Hall contribution to Hall conductivity spectra.

In this section, we discuss the ordinary Hall contribution to the Hall conductivity

spectra, which is neglected in the main text. The ordinary Hall spectrum $\sigma_{xy}^{N}(\omega)$ in the Drude model is given by,

$$\sigma_{xy}^{N}(\omega) = \frac{nq^2}{m}\frac{\omega_{\mathrm{c}}}{(\omega + i/\tau)^2 - \omega_{\mathrm{c}}^2} = \frac{\sigma_{xx}(0)\omega_c/\tau}{(\omega + i/\tau)^2 - \omega_{\mathrm{c}}^2}, \quad (2)$$

where m, n and q represent carrier mass, carrier density and electric charge, respectively. The DC conductivity $\sigma_{xx}(0)$ and scattering rate $1/\tau$ were determined by the transport measurement and Drude analysis of $\sigma_{xx}(\omega)$ spectra, respectively. The cyclotron frequency ω_{c}, which is given by $\frac{eB}{m}$, was calculated by combining $\sigma_{xx}(0)$, $1/\tau$ and n determined from the ordinary Hall effect. For example, we obtained the cyclotron frequency $\hbar\omega_{\mathrm{c}}$ = 2.3 μeV at 4.5 K, 7 T. The resultant ordinary Hall spectrum (Supplementary Fig. 5) should not show any significant structure in our measurement energy (0.7 - 6 meV).

Supplementary Note 3: Extension of theoretical model

As described in the main text, we consider the minimal model to discuss the spectral characteristics in $\sigma_{xy}(\omega)$ producing the AHE. The single anti-crossing point in the two-dimensional electronic structure is expressed by the following Hamiltonian $H(k)$,

$$H(k) = -\mu\sigma_0 + \sum_{i=x,y,z} h_i(k)\sigma_i\,, \quad (3)$$

where σ_0 and σ_i (i=x, y, z) are the identity and Pauli matrices, respectively, and μ is the chemical potential. Assuming that the band dispersion is two-dimensional and (h_x, h_y, h_z) = (k_x, k_y, m), the corresponding band dispersion has the level splitting of 2|m| at the crossing point, which is schematically illustrated in the inset of Fig. 3a in the main text.

We calculate the optical Hall conductivity $\sigma_{xy}(\omega)$ with use of the general

expression given by the Kubo formula;

$$\sigma_{xy}(\omega) = i\sum_{n,m}\frac{f(\varepsilon_m)-f(\varepsilon_n)}{\varepsilon_m-\varepsilon_n}\frac{\langle m|J_y|n\rangle\langle n|J_x|m\rangle}{\omega+i\delta+\varepsilon_m-\varepsilon_n} \qquad (4)$$

where the $J_{x(y)}$ is the current operator given by $\frac{\hbar}{e}\sum_k c^\dagger(k)\frac{\partial H(k)}{\partial k_{x(y)}}c(k)$, $f(\varepsilon_n)$ is the Fermi distribution function, ε_n and $|n\rangle$ are the energy and the Bloch wave function of the n-th band, respectively, and δ is the damping constant. The energy-dependent Hall conductivity $\sigma_{xy}(\omega)$ is thus given by[S7,S8];

$$\sigma_{xy}(\omega) = \frac{e^2}{2ha}\frac{m}{\hbar\omega+i\delta}\ln\left|\frac{-\hbar\omega-i\delta+2\mu}{\hbar\omega+i\delta+2\mu}\right|, \qquad (5)$$

where e, h, δ and a are the elementary electric charge, Planck constant, phenomenological broadening factor and lattice constant, respectively. The DC-limit value expected from Supplementary Eq. (5) is necessarily smaller than $\frac{e^2}{2ha}$. This value about ~ 400 $\Omega^{-1}\mathrm{cm}^{-1}$ for MnGe with a = 0.48 nm is well comparable with the intrinsic AH conductivity value of in the conventional ferromagnetic (Weyl) metals including the present case. Thus, to adjust this prefactor depending on the actual 3D band structure with dispersive gap magnitude m, we phenomenologically extend the above formula by introducing a free parameter $f_{\alpha,\beta}$ representing the spectral weight in Eq. (4) of the main text. We note that in MnGe with chiral crystal structure the band crossing with intense Berry curvature is the (three-dimensional) Weyl point, which is robust against subtle perturbation; the minimal Hamiltonian is described by, $H = \sum_{i=x,y,z} v_i\, k_i\sigma_i$. In the present model, we consider the anti-crossing points present along the certain k vector interconnecting two Weyl points (Fig. 3b, main text).

Supplementary Note 4: Estimation of mean free path of MnGe thin film.

In the free-electron model, the mean free path l can be given as,

$$l = \frac{\hbar}{ne^2}(3\pi^2 n)^{1/3}\sigma_0, \tag{6}$$

where n is the carrier density and σ_0 is the longitudinal conductivity. We calculated n from the ordinary Hall effect at 100 K and used σ_0 obtained in the transport measurement. As a result, we obtained l ~ 3.6 nm at 2 K.

Supplementary Note 5: First-principles calculation of the band structure and optical Hall conductivity spectra for the forced ferromagnetic state.

In this section, we show the first-principles calculations for the forced ferromagnetic state (Supplementary Fig. 6; see also ref. S9). Electronic structure calculations for ferromagnetic MnGe were performed using the density functional theory with a generalized gradient approximation[S10] as implemented in the quantum-ESPRESSO code[S11]. Ultrasoft pseudopotentials[S12] and the plane-wave basis set with cutoff energies of 50 Ry for wave functions and 400 Ry for charge densities were used. We observe a lot of band crossings between the high-symmetry k points and probably there should be much more crossing points in the general k space.

The optical Hall conductivity spectra were calculated by using Kubo-Greenwood formula given by,

$$\sigma_{\alpha\beta}(\hbar\omega) = \frac{ie^2\hbar}{N_k\Omega_c}\sum_{\boldsymbol{k},n,m}\frac{f_{m,\boldsymbol{k}} - f_{n,\boldsymbol{k}}}{\varepsilon_{m,\boldsymbol{k}} - \varepsilon_{n,\boldsymbol{k}}}\frac{\langle\psi_{n,\boldsymbol{k}}|v_\alpha|\psi_{m,\boldsymbol{k}}\rangle\langle\psi_{m,\boldsymbol{k}}|v_\beta|\psi_{n,\boldsymbol{k}}\rangle}{\varepsilon_{m,\boldsymbol{k}} - \varepsilon_{n,\boldsymbol{k}} - (\hbar\omega + i\eta)}, \tag{7}$$

where e, $\hbar$, Ω_c, N_k, η, $f_{n,\boldsymbol{k}}$ are the elementary charge with negative sign, reduced Planck constant, cell volume, number of k-point, smearing parameter, and the Fermi-Dirac

distribution function with the band index n and the wave vector $\boldsymbol{k}$, respectively. The $\sigma_{\alpha\beta}(\hbar\omega)$ was calculated using the Wannier-interpolated band structure with a 400 ×400×400 k-point grid and η = 1 meV. As shown in Supplementary Fig. 7, at the Fermi level E_F = -0.22 eV, for example, we observe the sharp resonance with positive sign, which well captures the spectral characteristics of the terahertz resonance producing the AHE and verifies our theoretical model assumed in the present analysis.

Supplementary Note 6: Growth and characterization of thin film.

We fabricated MnGe thin films by molecular beam epitaxy (MBE) method. We used a 2-nm-thick MnSi (111) buffer layer, which was grown by reacting a deposited Mn layer with the Si(111) surface at 250 °C. We then co-evaporated Mn and Ge at ~150 °C and subsequently annealed it at 250 °C. Supplementary Fig. 8 shows the x-ray diffraction (XRD) pattern for the film we used, which confirms the epitaxial growth of B20-type MnGe along <111> direction. In addition, some of our coauthors have already established that our thin film system does not include any other impurity phases by means of the transmission electron microscope (TEM) and the energy dispersive x-ray spectroscopy (EDX) in the previous study[S13]. The TEM image confirms the epitaxial growth of B20-type MnGe without any amorphous states (Fig. S2(a,b) in Supplemental Material of ref. 13) and the EDX mapping shows the homogeneous distributions of Mn and Ge elements (Fig. S2(c-f) in Supplemental Material of ref. S13).

We also compare the transport properties of the thin film used here and micro-size single crystal[S14] as shown in Supplementary Fig. 9; the size of the single crystal is ~ 10×10×3 μm^3 and therefore it hardly includes any impurity phases (see also ref. S5). The temperature dependence of the resistivity shows the good metallic behaviors both for the

single crystal and thin film, which are consistent with each other (Supplementary Fig. 9a,b). The comparison of the magnetic-field dependence of the Hall resistivity is shown in Supplementary Fig. 9c-f, where the overall features show the similar tendency. At high temperatures, we only observe the AHE and OHE. With decreasing the temperature, the magnetic-field dependence of the Hall resistivity shows the dip-like structure at the intermediate field regions, which corresponds to the THE. The magnitude of the AHE tends to be larger in the thin film case, which might be attributed to the strain effect on the film, while that of the THE is comparable.

Here we further discuss the following two points to corroborate that the terahertz spectra obtained here cannot be interpreted by spurious effects such as the parallel conduction of multiple carriers observed in Ge:Mn systems[S15,S16]. First, the terahertz conductivity spectra shown in Fig. 1f are well fitted by the single carrier Drude model, suggesting one dominant conduction channel rather than the parallel conduction. Second, the typical energy scale of the observed terahertz resonance is basically unchanged by the magnetic field as shown in Fig. 2a,b, in stark contrast to the cyclotron resonance, whose resonance energy is proportional to the magnetic field. Therefore, the clear terahertz resonance in $\sigma_{xy}(\omega)$ should be intimately associated with the AHE and/or THE of MnGe.

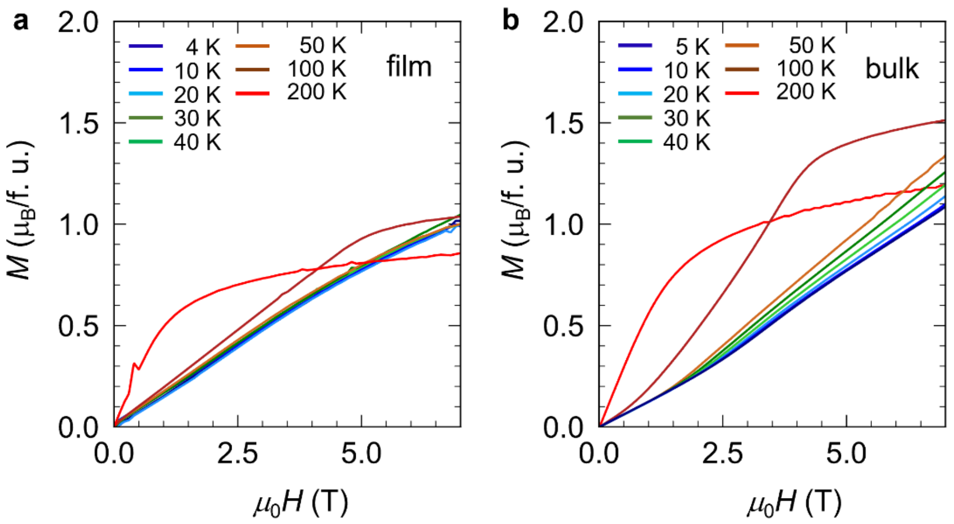

Supplementary Figure 1| Magnetization of MnGe. a,b, Magnetic-field dependence of the magnetization for (**a**) thin film and (**b**) bulk poly crystal[S2]. The film magnetization could be measured only up to 7 T.

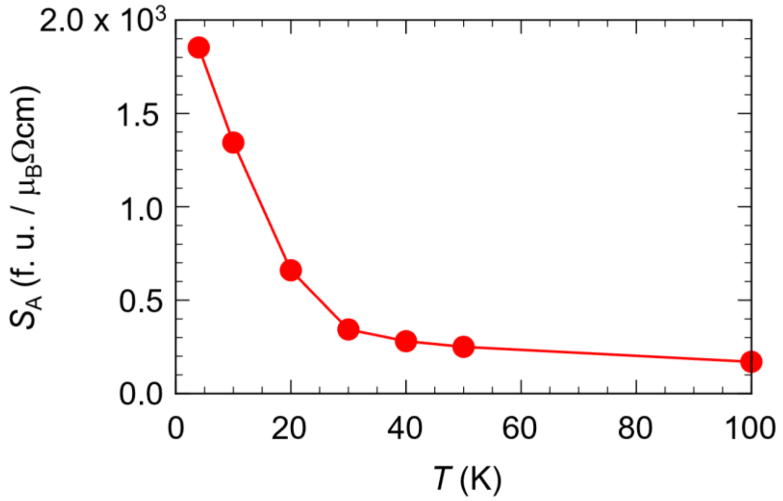

Supplementary Figure 2| The temperature dependence of anomalous Hall coefficient. The anomalous Hall coefficient S_A steeply increases with lowering the temperature, which is in accord with the observation of the sharp low-energy resonance feature of $\sigma_{xy}(\omega)$, as discussed in the main text.

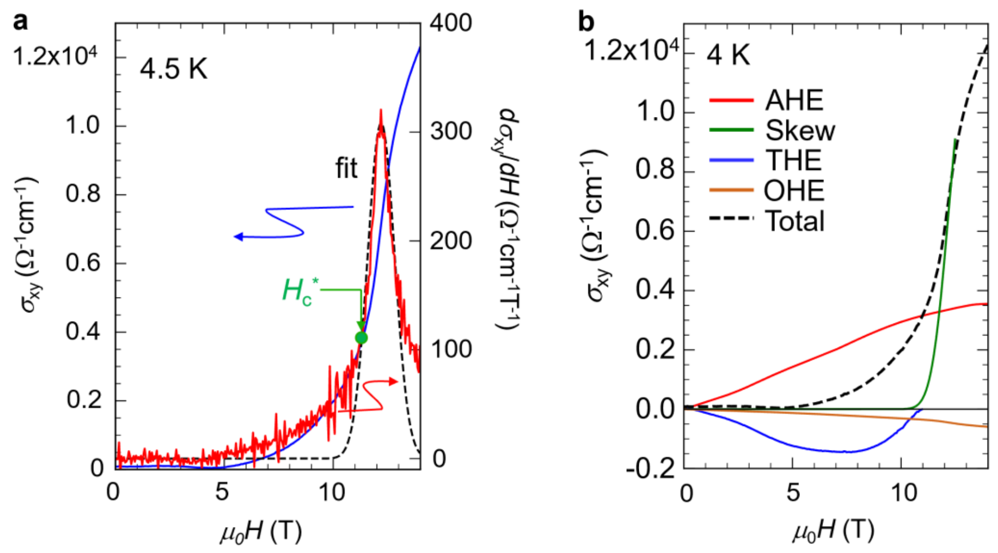

Supplementary Figure 3| Decomposition of different origins for the Hall conductivity. a, Magnetic-field dependence of the Hall conductivity σ_{xy} (blue curve) and corresponding $d\sigma_{xy}/dH$ (red curve). The dotted curve represents the Gaussian fitting of the peak anomaly in $d\sigma_{xy}/dH$, which shows the steep increase of σ_{xy} arising from the spin-chirality skew scattering. **b,** The decomposition of total σ_{xy} (dotted curve) into the anomalous Hall (red curve), topological Hall (blue curve), ordinary Hall (orange curve) and spin-chirality scattering components (green curve). Since the spin-chirality skew scattering should be enhanced near the H_C, the decomposition discussed here might be less accurate near the H_C. Nevertheless, the AHE and THE can be estimated appropriately in the low-field region (≤ 7 T) focused in this paper.

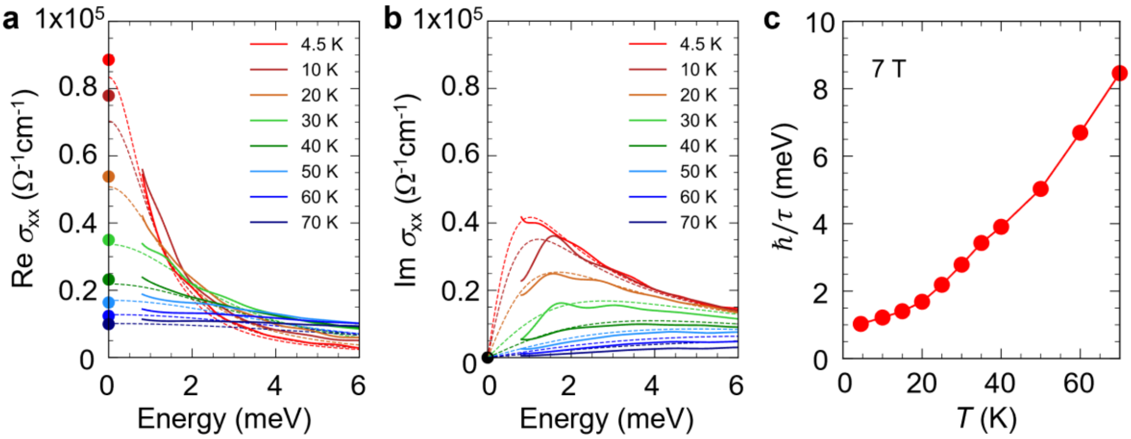

Supplementary Figure 4| Optical conductivity of MnGe thin film. a,b, The real (**a**) and imaginary (**b**) parts of the optical conductivity of MnGe thin film. The dashed lines are the fitting curves obtained from the Drude model. **c,** Temperature dependence of the scattering rate ($\hbar/\tau$) deduced from the Drude model analysis.

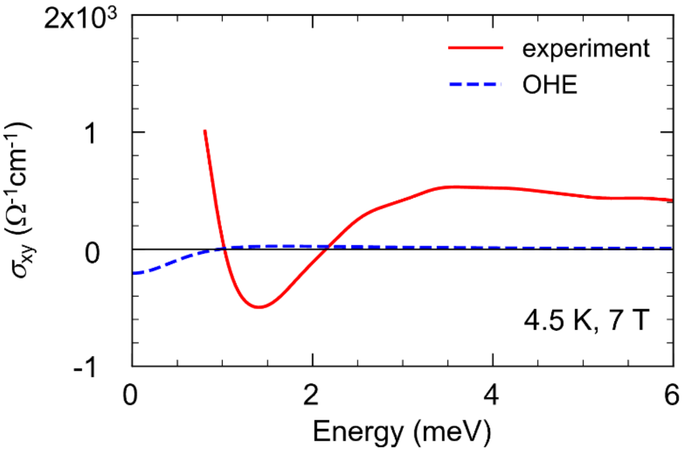

Supplementary Figure 5| Ordinary Hall conductivity spectrum. The Hall conductivity spectrum acquired from the experiment (red curve), and the calculated ordinary Hall conductivity spectrum (blue dashed curve).

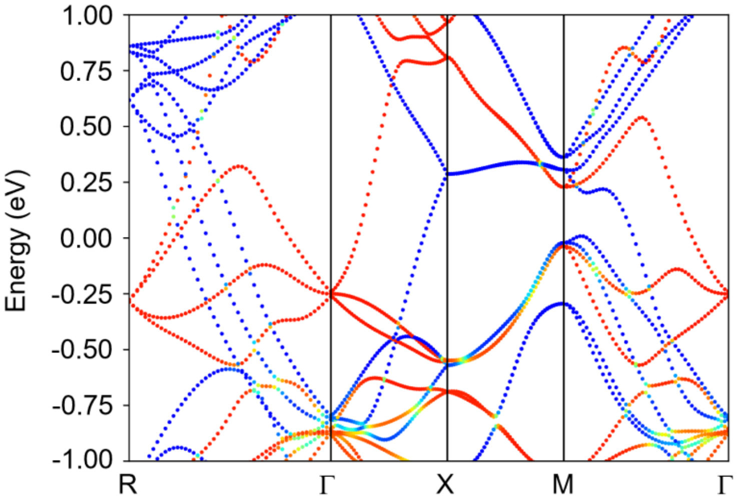

Supplementary Figure 6| Density functional theory calculation for the ferromagnetic state of MnGe. The band structure calculation for the ferromagnetic state. The reddish and bluish colors represent the up-spin and down-spin bands, respectively.

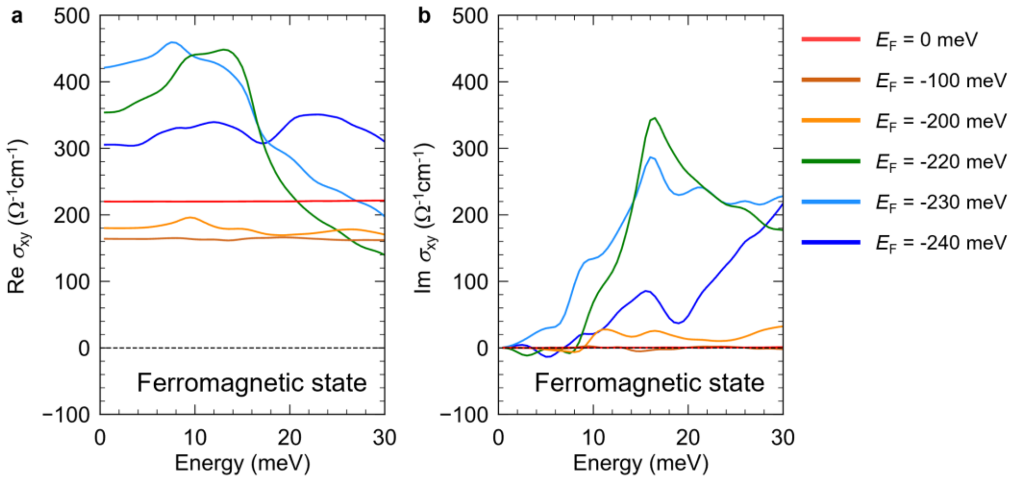

Supplementary Figure 7| Calculation of optical Hall conductivity spectra. a,b, The real (a) and imaginary (b) parts of the optical Hall conductivity spectra for the ferromagnetic state for various Fermi levels.

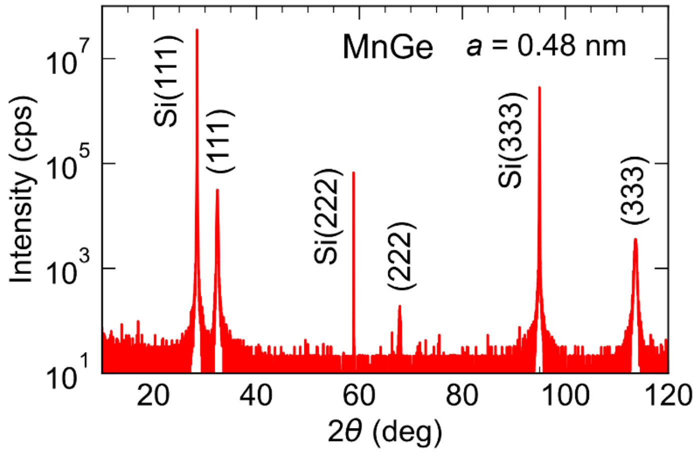

Supplementary Figure 8| θ-2θ x-ray diffraction pattern of MnGe thin film. All peaks can be indexed to the B20-type MnGe or Si substrate, suggesting that the MnGe thin film is single crystalline and oriented along [111] direction perpendicular to the substrate surface.

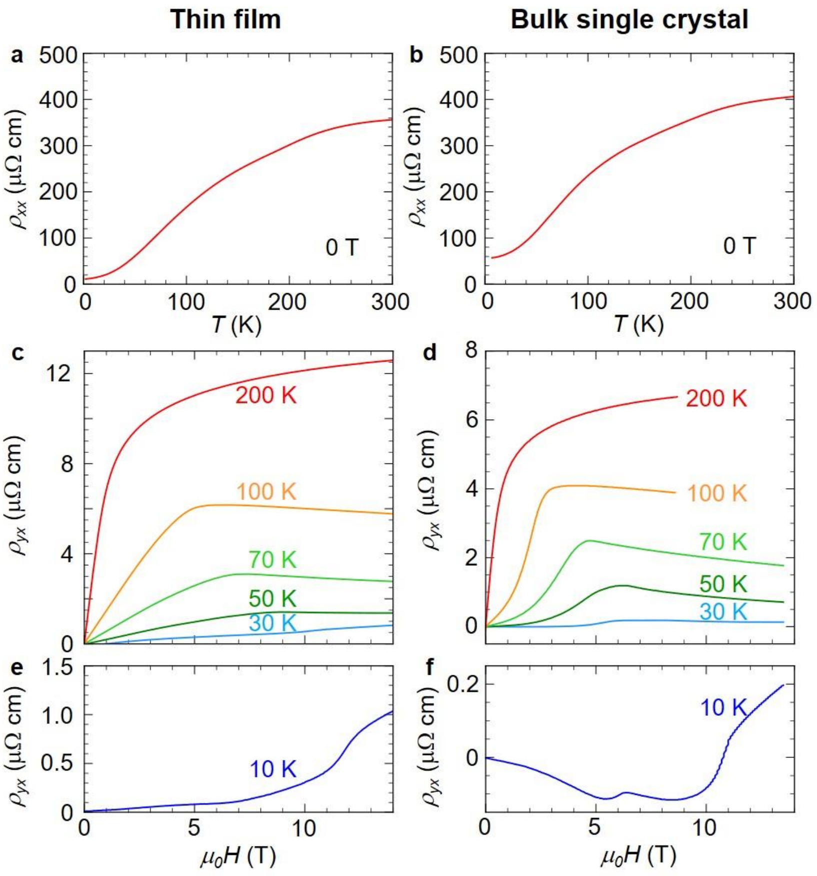

Supplementary Figure 9| Comparison of transport properties for the bulk single crystal and thin film. a,b, The temperature dependence of resistivity for single crystal (a) and thin film (b). **c-f,** The magnetic-field dependence of the Hall resistivity at each temperature for the single crystal (c,e) and thin film (d,f). The bulk single crystal data are reproduced from the work by Kitaori *et al*.[14]

Supplementary References

S1. Lee, M., Onose, Y., Tokura, Y., & Ong, N. P., Hidden constant in the anomalous Hall effect of high-purity magnet MnSi. *Phys. Rev. B* **75,** 172403 (2007).

S2. Kanazawa, N. *et al.* Large topological Hall effect in a short-period helimagnet MnGe. *Phys. Rev. Lett.* **106,** 156603 (2011).

S3. Aharoni, A., Demagnetizing factors for rectangular ferromagnetic prisms. *J. Appl. Phys*. **83,** 3432 (1998).

S4. Fujishiro, Y. *et al*. Giant anomalous Hall effect from spin-chirality scattering in a chiral magnet. *Nat. Commun.* **12,** 317 (2021).

S5. Kanazawa, N. *et al.* Direct observation of the statics and dynamics of emergent magnetic monopoles in a chiral magnet. *Phys. Rev. Lett.* **125,** 137202 (2020).

S6. Ishizuka, H., & Nagaosa, N., Spin chirality induced skew scattering and anomalous Hall effect in chiral magnets. *Sci. Adv.* **4,** eaap9962 (2018).

S7. Iguchi, S. *et al.* Optical probe for anomalous Hall resonance in ferromagnets with spin chirality. *Phys. Rev. Lett.* **103,** 267206 (2009).

S8. Shimano, R. *et al.* Terahertz Faraday rotation induced by an anomalous Hall effect in the itinerant ferromagnet $SrRuO_3$. *Europhys. Lett.* **95,** 17002 (2011).

S9. Fujishiro, Y. *et al.* Large magneto-thermopower in MnGe with topological spin texture. *Nat. Commun.* **9,** 408 (2018).

S10. Perdew, J. P., Burke, K. & Ernzerhof, M. Generalized gradient approximation made simple. *Phys. Rev. Lett.* **77,** 3865-3868 (1996).

S11. Giannozzi, P. *et al*. Quantum ESPRESSO: a modular and open-source software project for quantum simulations of materials. *J. Phys. Condens. Matter* **21,** 395502 (2009).

S12. Vanderbilt, D. Soft self-consistent pseudopotentials in a generalized eigenvalue formalism. *Phys. Rev. B* **41,** 7892 (1990).

S13. Kanazawa, N. *et al.* Topological spin-hedgehog crystals of a chiral magnet as engineered with magnetic anisotropy. *Phys. Rev. B* **96,** 220414(R) (2017).

S14. Kitaori, A. *et al.* Enhanced electrical magnetochiral effect by spin-hedgehog lattice structural transition. arXiv.2104.12120 [cond-mat.str-el].

S15. Zou, S. *et al.* Anomalous Hall resistance in Ge:Mn systems with low Mn concentrations. *Appl. Phys. Lett.* **95,** 172103 (2009).

S16. Simons, A. *et al*. Components of strong magnetoresistance in Mn implanted Ge. *J. Appl. Phys.* **115,** 093703 (2014).
